# A metal-organic cage incorporating multiple light harvesting and catalytic centres for photochemical hydrogen production

**DOI:** 10.1038/ncomms13169

**Published:** 2016-11-09

**Authors:** Sha Chen, Kang Li, Fang Zhao, Lei Zhang, Mei Pan, Yan-Zhong Fan, Jing Guo, Jianying Shi, Cheng-Yong Su

**Affiliations:** 1 MOE Laboratory of Bioinorganic and Synthetic Chemistry, State Key Laboratory of Optoelectronic Materials and Technologies, Lehn Institute of Functional Materials, School of Chemistry and Chemical Engineering, Sun Yat-Sen University, Guangzhou 510275, China; 2 State Key Laboratory of Organometallic Chemistry, Shanghai Institute of Organic Chemistry, Chinese Academy of Sciences, Shanghai 200032, China

## Abstract

Photocatalytic water splitting is a natural but challenging chemical way of harnessing renewable solar power to generate clean hydrogen energy. Here we report a potential hydrogen-evolving photochemical molecular device based on a self-assembled ruthenium–palladium heterometallic coordination cage, incorporating multiple photo- and catalytic metal centres. The photophysical properties are investigated by absorption/emission spectroscopy, electrochemical measurements and preliminary DFT calculations and the stepwise electron transfer processes from ruthenium-photocentres to catalytic palladium-centres is probed by ultrafast transient absorption spectroscopy. The photocatalytic hydrogen production assessments reveal an initial reaction rate of 380 μmol h^−1^ and a turnover number of 635 after 48 h. The efficient hydrogen production may derive from the directional electron transfers through multiple channels owing to proper organization of the photo- and catalytic multi-units within the octahedral cage, which may open a new door to design photochemical molecular devices with well-organized metallosupramolecules for homogenous photocatalytic applications.

## Abstract (web summary)

Photocatalytic water splitting is a promising route to hydrogen generation from renewable solar power. Here, the authors report a hydrogen-evolving photochemical molecular device based on a self-assembled coordination cage, which simultaneously incorporates multiple photosensitizing and catalytic metal centres.

## Graphical abstract

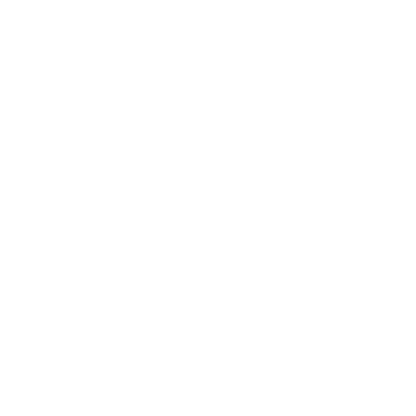

Visible light-driven water splitting to produce hydrogen is now considered as an attractive alternative energy source leading to solar energy conversion and storage. In this context, crystalline metal-organic frameworks (MOFs) have been widely studied in photocatalytic water splitting in heterogeneous conditions^1^,^2^,^3^ owing to the advantages that verstaile organic chromophores and catalytically active metal centres can be integrated into the framework, or, loaded into the pores. In contrast, the analogous metal-organic cages/containers (MOCs), which feature discrete, nanoscale metallosupramolecules with well-designed shape and size have drawn little attention for photocatalytic H_2_ production, bearing in mind that MOCs have been vigorously investigated for many other reactions^4^,^5^,^6^,^7^,^8^,^9^ in terms of the confined coordination space and guest-selective windows, especially with relevance to enzyme mimics. In addition to the MOF-like advantages such as assembly of multiple, functional organic ligands and metal centres, MOCs also merit consideration because they may be able to organize the active subcomponents in a specific fashion to achieve collaborative and synergistic functions reminiscent of photosystems I and II (ref. 10), and accomplish catalysis in homogeneous conditions.

As long as the photochemical H_2_ evolution has been considered, semiconductor photocatalysts have been studied in heterogeneous catalytic systems^11^. Meanwhile, molecule-based three-component photosystems also attracted extensive attention in homogenous photocatalysis since the 1970s (ref. 12). Remarkable progress has been made since the development of intramolecular photochemical molecular devices (PMDs) by integrating the chromophoric photosensitizer, catalytic centre and electron relay components into a single-component photocatalyst^13^,^14^,^15^. Compared with the three-component intermolecular photocatalytic systems^16^,^17^,^18^, the covalently or coordinatively linked single-component intramolecular PMDs are expected to improve the overall catalytic efficiency by avoiding the molecular collision before relaxation of the ^3^MLCT excited state, therefore affording efficient electron and energy transfer^12^,^19^,^20^,^21^,^22^.

To date, many active bimetallic PMDs, such as Ru–Pt^12^,^14^,^21^, Ru–Pd^13^,^23^,^24^,^25^, Ru–Rh^26^, Ru–Co^15^ and Ir–Co^27^ complexes, have been developed for homogenous catalysis. Considering the multiple electrons transfer and accumulation required for H_2_ evolution, many efforts are made to improve the effective and directional electron transfer from light absorbing centres to catalytic metal centres^28^,^29^,^30^. The main strategies include modification of bridging and/or peripheral ligands at the photocentre, optimization of co-ligand at the catalytic centre^31^,^32^, as well as design of self-assembled polynuclear systems^33^,^34^,^35^. Another promising approach to improve catalytic performance is to incorporate more photosensitizing and/or catalytic units within a single PMD. Brewer and co-workers developed the active trinuclear Ru–Rh–Ru and tetranuclear Ru_2_–Ru–Pt complexes^32^,^36^ comprising two and three photocentres, respectively, achieving 870 turnover number (TON) after 46 h. Sakai also accomplished higher H_2_ production by preparing trinuclear Ru–Pt_2_ and tetranuclear Ru–Pt_3_ complexes introducing two and three reactive centres, respectively, in comparison with the dinuclear Ru–Pt analogue^12^. Moreover, a decanuclear OsRu_3_Pt_6_ complex with dendrimeric chromophores was found to exhibit a remarkable antennae effect, where all the absorbed energy is efficiently transferred to a single Os unit^37^, although no test for multi-electron photocatalysis was performed. Nevertheless, simply increasing the metal nuclearity in a single PMD based on covalent multi-metallic complexes may lead to undirectional electron transfer and tedious separation of various geometric and optical isomers^22^. Therefore, report on efficient PMDs simultaneously incorporating multiple photosensitizers and catalytic centres remains rare.

Herein we present a PMD model based on a self-assembled Ru_8_–Pd_6_ MOC^38^,^39^, in which eight Ru^2+^-photocentres and six catalytically active Pd^2+^-centres are organized in a highly ordered octahedral manner to provide multiple but independent energy transfer and electron collection pathways (Fig. 1). The photocatalytic H_2_ production tests reveal a high TON of 635 in 48 h and prolonged photocatalytic robustness. To the best of our knowledge, such a self-assembled MOC as the single hydrogen-evolving PMD made up of multiple photo- and catalytic metal centres has not been reported yet, although Duan *et al*.^40^ have utilized MOCs for photocatalytic H_2_ generation via encapsulation of organic dyes.

## Results

### PMD structural model

**Figure 1:**
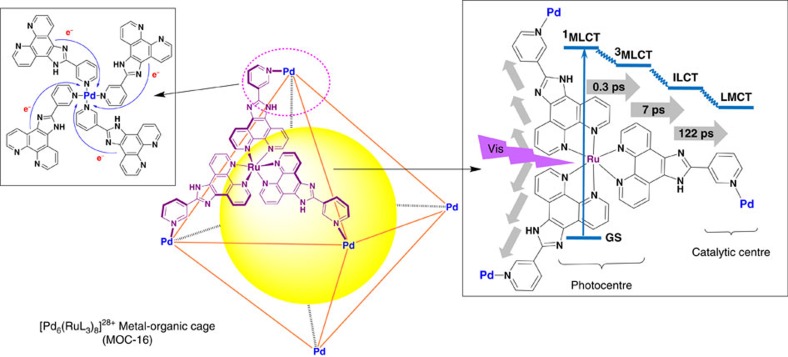
PMD structural model. Octahedral cage structure of [Pd_6_(RuL_3_)_8_]^28+^ and the multi-channel electron transfer pathways between chromophoric Ru and catalytic Pd metal centres. GS, ground state; ILCT, intraligand charge transfer; LMCT, ligand-to-metal charge transfer; MLCT, metal-ligand charge transfer.

The heteronuclear [Pd_6_(RuL_3_)_8_]^28+^ coordination cage (MOC-16, Fig. 1) can be readily obtained from an one-pot reaction of the predesigned redox- and photoactive RuL_3_ metalloligands (L=2-(pyridin-3-yl)-1H-imidazo[4,5-f][1,10]-phenanthroline) and Pd^2+^ ions as previously reported (Supplementary Note 1)^38^,^39^, showing the shape of octahedron with six Pd^2+^ atoms on the vertices and eight Ru^2+^ atoms on the facets. The ligand L consists of a phenanthroline (Phen) part and an benzimidazole-pyridine (BIm–Py) part, playing the dual role of bridging and peripheral ligands in normal one-component bimetallic PMDs. The solution dynamics study has confirmed that MOC-16 is the sole thermodynamically stable product in solution, where appearance of one set of ^1^H NMR pattern is indicative of high *O*-symmetry for the [Pd_6_(RuL_3_)_8_]^28+^ cage^38^,^39^. Such an octahedron-shaped Ru_8_–Pd_6_ PMD structure implies that the eight Ru(Phen)_3_ photosensitizers and the six Pd(Py)_4_ catalysers are individually equivalent, while spatially separated and fixed, facilitating independent and directional electron transfers without unnecessary mutual electronic coupling^12^. Therefore, effective multi-channel electron transfer may be achieved in this kind of self-assembled and well-organized polynuclear PMDs (Fig. 1).

### Photocatalytic hydrogen production

**Figure 2:**
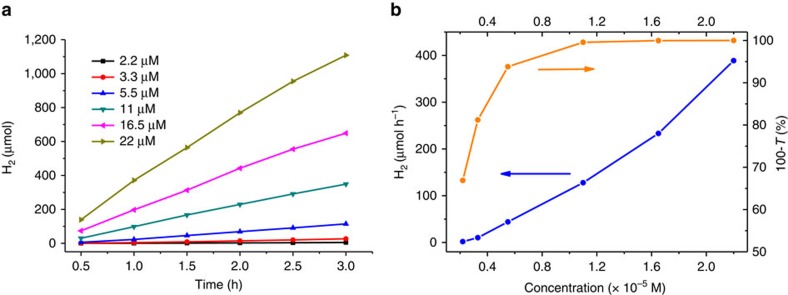
H_2_ evolution tests. (**a**) H_2_ evolution with variable MOC-16 concentrations in DMSO solution containing 0.34 M H_2_O and 0.75 M TEOA. (**b**) Dependence of the photoabsorption completeness (orange, *T* denotes the mean transmittance) and the H_2_-production rate (blue) on the concentration of MOC-16.

The photocatalytic evolution of H_2_ in the presence of MOC-16 are undertaken in a closed gas circulation and evacuation system (Supplementary Fig. 1) irradiated with visible light (>420 nm, Supplementary Note 2). Since MOC-16 is well soluble in dimethylsulfoxide (DMSO) while moderately soluble in water, we choose aqueous DMSO mixture as the reaction solution to study its photocatalytic performance. Figure 2a gives 3-hour H_2_ evolution tests at various MOC-16 concentrations in a DMSO solution containing 0.34 M H_2_O and 0.75 M TEOA. It is clear that, at each MOC-16 concentration, H_2_ production increases almost linearly by prolonging the irradiation time. The photoabsorption completeness and the H_2_ production rate are plotted as a function of the MOC-16 concentration in Fig. 2b. We see that the photoabsorption gradually increases with the MOC-16 concentration increasing from 2.2 to 11 μM, where the platform of 100% absorption is achieved.

**Figure 3:**
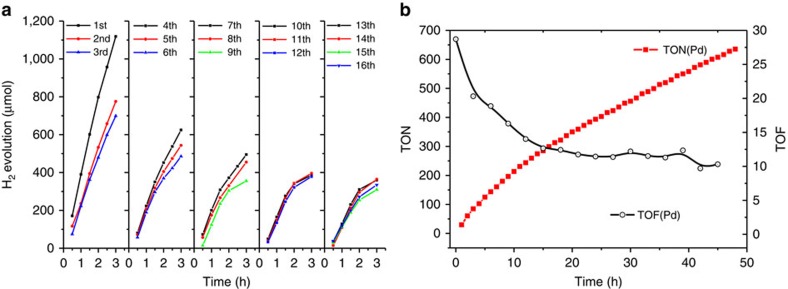
Long-term H_2_ evolution. (**a**) H_2_ production of consecutive 16 3-h runs. (**b**) Accumulated TONs and TOFs based on Pd-centre in catalyst durability test over 48 h. TON=*n*(H_2_)/*n*(Pd), TOF=*d*_(TON)_/*d*_t_.

The catalyst durability test is carried out under the optimized conditions (100 ml DMSO solution with 22 μM MOC-16, 0.34 M H_2_O and 0.75 M TEOA) taking into account the utilization of the full photoabsorption and the relatively high H_2_ evolution rate. Figure 3a shows H_2_ evolution as a function of time in consecutive 16 reaction cycles, 3 h in every run and 48 h in total (16 × 3 h). Albeit the H_2_ evolution dependence on time deviates from linearity gradually in later runs, the initial rate in every run can be evaluated from the slope of straight line portion at the beginning (Supplementary Fig. 2). The highest rate of 380 μmol h^−1^ appears in the first run, and then declines gradually to *ca.* 150 μmol h^−1^. Figure 3b presents the accumulated TONs (based on Pd centres) and apparent turnover frequencies (TOFs) over 48 h irradiation, in which TOF is calculated as *d*_(TON)_/*d*_t_, known as turnover rate referred to the number of catalytic sites and relevant to the initial rate^41^. It is evident that TON increases steadily along reaction time and reach to 635 in 48 h. The maximum TOF of 30 h^−1^ also appears in the first run, decreasing gradually to *ca.* 11 h^−1^ with prolonged irradiation time. The simultaneous dropping of TOF and initial rate of H_2_ production along the multiple photocatalytic trials is contrary to other Ru–Pd systems^24^,^29^, where TOF usually increases and fluctuates significantly along reaction at early time.

The long-term reactivity and high initial rate for H_2_ evolution photocatalysed by MOC-16 are surprising considering that the catalytic centres in MOC-16 are Pd(Py)_4_ motifs which possess a PdN_4_ environment with four monodentate pyridyl donors. For the known PMDs^12^,^19^,^20^,^21^,^22^, the N̂N-chelated N_*n*_MX_2_ (M= Pd^2+^, Pt^2+^ or Rh^2+^, X= Cl^−^, Br^−^ or I^−^, *n*=2 or 4) coordination sphere was commonly adopted as a catalyst centre, for which dissociation of the terminal X^−^ anions during the photolysis was also pointed out^24^,^25^. Moreover, some of the works revealed that colloidal Pd has a major contribution to the photoinduced H_2_ evolution in such a Ru–Pd supramolecular system^42^. In the present case, we did observe the formation of black Pd-particles after a long-time irradiation of the photocatalyst solution (*ca.*100 h), which was confirmed by transmission electron microscopy measurements to show clear lattice fringes of Pd (111) and (200) crystal faces (Supplementary Fig. 3). However, the photocatalytic behaviour of MOC-16 is relatively complicated in comparison with those previously reported for other PMDs (Supplementary Figs 3–6).

To check the contribution of Pd-particles to H_2_ production, a control experiment was performed by directly mixing free RuL_3_ metalloligand with the Pd-particles, where commercial Pd-black or Pd-particles, *in situ* generated by ultraviolet irradiation of Pd(BF_4_)_2_ in DMSO overnight, were used as the Pd-source. In both conditions, only trace amount of H_2_ was detected (Supplementary Fig. 4). These results support that the formation of Pd-particles is not essential for the photocatalytic H_2_ evolution activity of MOC-16. We rather consider that the relatively robust octahedral cage structure is responsible for the highest catalytic performance observed at the initial stage of photolysis. This behaviour is in sharp contrast with those previously reported for the Ru–Pd systems^24^,^29^, in which a clear induction period was observed in H_2_ evolution profiles. To check if Pd-particles are formed at early reaction stage and elucidate its role in the H_2_ evolution photocatalysed with MOC-16, another control experiment was carried out by comparing two parallel reactions, which were alternately performed with each comprising 12 cycles of 3-hour reaction (12 × 3 h) in total 36 h (see more details in Supplementary Note 3). One reaction proceeded consecutively without any treatment of the reaction mixture. The other was treated at reaction intervals in two ways: (i) the reaction mixture was centrifugated after every 3-hour cycle in the early 24 h and; (ii) catalyst poisoning by mercury-drop was performed in the later 12 h (Supplementary Fig. 6). The results reveal that no Pd-particles are detectable in the first 24 h, and the presence of 100-fold excess Hg shows negligible poisoning effect on the H_2_ production activity of MOC-16 in the final 12 h photolysis stage. These results ascertain that the photocatalytic performace of MOC-16 does not rely on the formation of Pd-particles but is well attributable to the cage structure composed of multiple Ru(Phen)_3_ and Pd(Py)_4_ moieties.

The prolonged reactivity and high TOF may corelate with the good thermodynamic stability of MOC-16 in solution as elucidated early^38^,^39^. Moreover, integrating six catalytic Pd centres in a whole cage may endow them with synergistic durability on structural deformation during catalysis on a Pd-centre, where dissociation of Pd–N bond and formation of hydride intermediate may occur^12^,^21^,^23^,^24^,^25^. The stability of MOC-16 is also confirmed by an Hg-test, where the ^1^H NMR spectra of MOC-16 recorded before and after the treatment with excess Hg in DMSO for 9 h shows no destruction of MOC-16 (Supplementary Fig. 7), while similar Hg-test was found to destroy the N_2_PdCl_2_ catalytic centre in a Ru–Pd PMD^24^. The robustness of the cage motif during the H_2_ production is further verified by a control experiment with a combination of separate [Ru(bpy)_3_]^2+^ (bpy—bipyridine) and [Pd(Py)_4_]^2+^ under similar conditions (Supplementary Fig. 5), which unveils that the unassembled [Pd(Py)_4_]^2+^ catalyst deactivates quickly in 2 h. These results implies a distinctive photocatalytic behaviour of MOC-16 in contrast to known Ru–Pd PMDs.

### Absorption and photoluminescence spectroscopy

**Figure 4:**
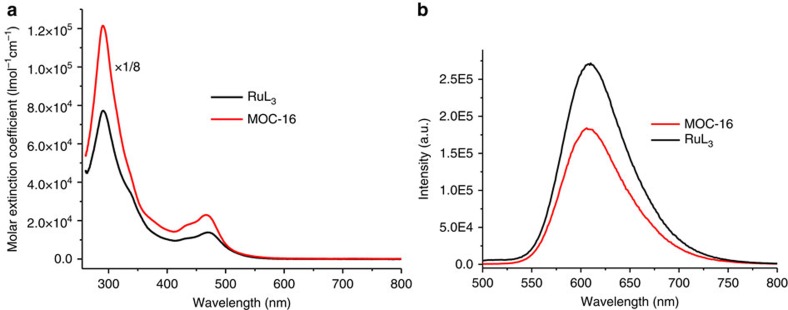
UV–VIS absorption and emission spectra of RuL_3_ metalloligand and MOC-16 in DMSO. (**a**) Electronic absorption spectra (*C*_RuL3_=8.0 × 10^−6^ and *C*_MOC-16_=1.0 × 10^−6^ M for equivalent RuL_3_). (**b**) Emission spectra (C_RuL3_=1.76 × 10^−4^ and C_MOC−16_=1.32 × 10^−5^ M for equal absorbance at excitation wavelength 466 nm).

In photocatalytic reaction, the energy and electron transition processes occur before hydrogen generation in catalytic centres. Therefore, it is necessary to study the photophysical properties of MOC-16 to further unveil the photocatalytic behaviours of H_2_ evolution. The UV-VIS (ultraviolet–visible) absorption and emission spectra of MOC-16 in DMSO, together with those of the free RuL_3_ metalloligand, are shown in Fig. 4. Since RuL_3_ moieties represent the main chromophores and luminophores, the concentration of MOC-16 was fixed at one-eighth of free metalloligand to give rise to equivalent RuL_3_ in absorption spectra, but was relatively diluted in emission measurement to have the equal absorbance with free RuL_3_ at the same excitation wavelength to compare their emission intensity by absorbing the same photon flux (Supplementary Fig. 8). It is clear that MOC-16 shows similar absorption and emission profiles with RuL_3_, indicating that introduction of Pd^2+^ does not significantly alter the ground-state spectroscopic property and substantially perturbe the energy gap between the lowest excited-state and ground-state^28^. A strong intraligand *π*–π* transition at 290 nm in ultraviolet region and a broad metal-to-ligand charge transfer (^1^MLCT of Phen←Ru) in 400–550 nm visible region are observed, in which the molar absorptivity is enhanced at every wavelength with negligible contributions from Pd(Py)_4_ units, as verified by comparison with UV–VIS absorption spectrum of free [Pd(Py)_4_]^2+^ salt at equivalent concentration (Supplementary Fig. 8), suggesting formation of a good light absorber by assembly of multiple chromophoric RuL_3_ units and absence of significant electronic communication between Ru-centres^21^. In contrast, the emission band at *ca*. 610 nm, which represents the Ru(Phen)_3_-centred triplet ^3^MLCT state, shows somewhat quenching effect after Pd^2+^-coordination. Compared with that of RuL_3_, the emission intensity of MOC-16 is reduced by about 32%, indicating a possible shift of the excited-state equilibrium from the radiative decay towards the radiationless deactivation via the intramolecular charge transfer to Pd(Py)_4_ moieties for catalysis^30^. This speculation is also relevant to the relaxation of the long-lived ^3^MLCT states in nanosecond domain (Supplementary Fig. 8; Supplementary Table 1), in which the PL decay of the lowest ^3^MLCT shows slight lifetime decrease from 601 (RuL_3_) to 484 ns (MOC-16) in DMSO. Such influence on long-lived states may relate to additional de-excitation channels in MOC-16 (electron transfer from the Ru chromophore to the terminal Pd(Py)_4_ moiety)^30^, but nevertheless insignificant since Pd-centre is far from Ru-photocentre and other deactivation reasons should not be excluded (*vide infra*)^28^.

### DFT calculation and electrochemistry

A preliminary calculation based on the structural model of the single-crystal^38^,^39^ has been performed with B3LYP hybrid functions (Supplementary Note 4) to disclose the electronic structure of MOC-16, which dominates its photophysical and photochemical properties. Due to the fact that the whole MOC-16 cage molecule is too large and a full calculation of this structure model is tremendously time-consuming, herein we only focus on the distribution of the related frontier orbitals. As shown in Supplementary Table 2, there are eight degenerated highest occupied molecular orbitals (HOMO to HOMO-7), which are mainly contributed by the *d*-orbitals of Ru^2+^-centres. On the other hand, the six degenerated lowest unoccupied molecular orbitals (LUMO to LUMO+5) comprise major contributions from the π* orbitals of pyridyl rings coordinated to Pd^2+^. Moreover, from LUMO+10 to LUMO+6, contributions for hybrid MOs can be found to spread over π* orbitals of different parts of Phen–BIm–Py ligand and move continually from the Ru-connecting Phen part to the bridging BIm moiety, and finally to the Pd-connecting Py part. Therefore, it is reasonable to postulate a photoinduced electron transfer pathway as illustrated in Fig. 1 and Supplementary Fig. 9 for the present system. Similar electron transfer in a Ru–Pt system based on the DFT calculation was also reported by Sakai *et al*.^21^,^43^.

The cyclic voltammogram (CV) curves of MOC-16 and RuL_3_ are shown in Supplementary Fig. 10 in together with those of free ligand L and [Pd(Py)_4_](BF_4_)_2_ as references, and the electrochemical data are collected in Supplementary Table 3. The MOC-16 displays a quasi-reversible apparent redox couple without splitting at +0.92 V (versus Fc/Fc^+^), which is assignable to Ru^III/II^ couple based on the similar oxidation potential of free RuL_3_ at +0.90 V (versus Fc/Fc^+^), closely comparable to other reported multiple Ru-systems^13^,^21^,^25^,^26^,^32^,^36^. The overlap of Ru^III/II^ couple indicates that eight Ru-centres in MOC-16 are electronically uncoupled in absence of significant electrochemical communications^42^,^43^,^44^,^45^, consistent with the spectra observation. This electrochemical result is further supported by above preliminary DFT calculations that the degenerated HOMOs are mainly based on Ru^2+^-centres, confirming that the eight Ru(Phen)_3_ photosensitizers in MOC-16 are individually equivalent to facilitate independent and directional electron transfers. In the negative potential window, RuL_3_ gives two irreversible reductions at −2.15 and −2.57 V, which are comparable to the reductions of free L ligand at −2.06 and −2.74 V. By contrast, MOC-16 shows three irreversible reductions at −1.58, −1.95 and −2.38 V (Supplementary Fig. 10 and Supplementary Table 3). The two peaks in the more negative region may be tentatively attributed to the ligand bound to Ru^2+^ and Pd^2+^, while the first irreversible reduction might relate to Pd(Py)_4_ moieties in MOC-16. To reveal the nature of this reduction, CV of a free [Pd(Py)_4_]^2+^ analogue was measured under resembling conditions, which gives an irreversible reduction peak at −1.04 V. In previous reports, negative shift of the Pd^2+^-reduction potential was observed from Pd(Py)_4_ (−1.365 V) to Pd(4-EtPy)_4_ (−1.475 V, versus Fc/Fc^+^) owing to *σ*-electron donation increase^46^, and irreversible reduction of Pd^2+^ was also reported for two Ru–Pd systems (−1.61 and −1.35 V, versus Fc/Fc^+^)^13^,^25^,^42^. In our case, the first reduction appears at much negative position in comparison with free [Pd(Py)_4_]^2+^ analogue (−1.58 versus −1.04 V), suggesting a strong hybridization of L ligands and Pd^2+^-centres, in agreement with above DFT calculations that the degenerated LUMOs spread over the pyridyl rings ligated to Pd^2+^. The broad feature of the first reduction peak implies potential electrochemical communication among the ligand-based reduction sites. However, it seems also probable that the reduction potentials of the ligand L show positive shifts upon coordination to Pd^2+^ (refs 21, 43).

### Ultrafast transient absorption

**Figure 5:**
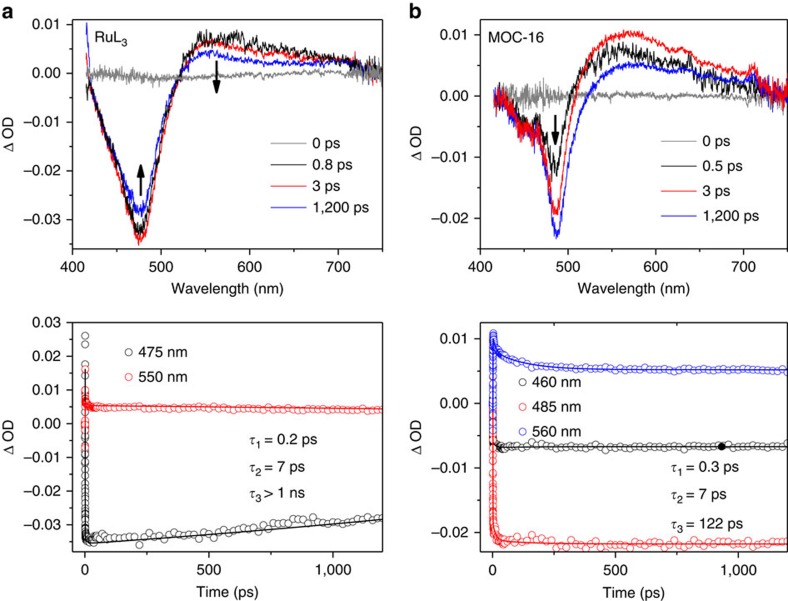
Transient absorption (TA) spectra excited at 400 nm and corresponding kinetic traces at selected wavelengths. (**a**) RuL_3_ and (**b**) MOC-16 in DMSO.

To better understand the photocatalytic processes, the excited-state dynamics in the picosecond time scale were investigated by ultrafast transient absorption spectroscopy, which is a powerful tool to clarify the electron transfer processes in PMDs^25^,^28^,^47^,^48^. On photoexcitation of the Ru chromophore at 400 nm, the transient absorption spectra of RuL_3_ and MOC-16 in DMSO at several delay times between pump and probe pulses are obtained, together with the kinetic traces (ΔOD versus time) at several key wavelengths (Fig. 5). More detailed kinetic traces over the first 25 ps at different probe wavelengths are shown in Supplementary Fig. 11. For RuL_3_, a broad excited-state absorption (ESA) band in 520–750 nm region is quasi-instantaneously generated. In addition, a strong structureless ground-state bleaching (GSB) band centred at 475 nm is observed. Such transient absorption profile of RuL_3_ is similar with those of reported [(bpy)_2_Ru(tpy)]^2+^ (tpy—2,2′:5′,2″-terpyridine) complex^28^ and dinuclear Ru–Co complex^45^. A global fitting transient absorption decay profiles at key wavelengths reveals that the dynamics of RuL_3_ may be properly described by two picoseconds components of 0.2 and 7 ps, as well as an extra long-lived component (>1,000 ps). For comparison, the transient absorption spectra of MOC-16 exhibit a broad ESA signal in 500–750 nm region accompanied by two discernable GSB signals centred at 460 and 485 nm. The splitting of GSB in MOC-16 should originate from the coupling of RuL_3_ with Pd^2+^, which alters excited electronic structure of the bridging L ligand^48^. It is worthy of noting that the GSB at 485 nm distinctly increases within 3 ps, showing no decay in the time scale from 0.5 to 1,200 ps. This indicates that the MOC-16 in the excited state does not simply deactivate to give the ground state but rather participates in the subsequent electron transfer processes (Supplementary Fig. 9). The global fitting of kinetic traces at key wavelengths reveals that three picoseconds components are needed to adequately describe the dynamics of MOC-16 in DMSO (0.3, 7 and 122 ps), which are different from the dynamics of free RuL_3_ as listed in Supplementary Table 1.

## Discussion

It has been well-established that the photoinduced dynamics in dinuclear PMDs, such as Ru–Pd^19^,^20^,^21^,^22^,^30^, Ru–Pt^21^,^23^, Ru–Co^49^ and Ru–Os^43^,^44^, feature in multi-step-relaxation processes^28^,^48^, involving intersystem crossing (ISC) from ^1^MLCT to ^3^MLCT^23^,^24^,^25^,^28^, intraligand charge transfer (ILCT)^23^,^24^,^25^,^28^,^48^, ligand-to-metal charge transfer (LMCT) and so on^23^,^24^,^25^,^28^,^48^. The electron transfer processes usually involve two MLCT states with excited electrons populating either different ligands (peripheral and bridging)^28^ or different parts of the ligands^23^,^24^,^25^,^43^,^44^, and result in electron transfer from excited states of photocentre to catalytic centre to form a charge-separted state^22^,^34^,^43^. In the present case, each Ru^2+^-centre is chelated by three identical L ligands which further link to three Pd^2+^-ions, and thus formed RuL_3_ metalloligands and Pd^2+^ ions are self-assembled into a multinuclear [Pd_6_(RuL_3_)_8_]^28+^ PMD, giving an octahedral MOC-16 cage with every Ru-photocentre, Pd catalyst centre and L ligand individually equivalent and spatially separated. Therefore, the photodynamic processes in such a well-organized polynuclear PMD are symmetrically unified, leading to uniform multi-channels for directional electron transfer as depicted in Fig. 1. This interpretation is supported by the results of our electrochemical and spectroscopic studies together with our preliminary DFT results. Formation of the long-lived excited states in MOC-16 may be considered to involve the ^3^MLCT states centred on the Phen motif and the ILCT state localized mainly on the BIm motif (Supplementary Fig. 9), similar to reported Ru–Pd(Pt) systems^23^,^24^,^25^,^28^,^30^.

In light of the above results from steady-state and transient spectra, electrochemistry and DFT calculations, the stepwise light-induced excited-state relaxation processes in MOC-16 may be tentatively illustrated in Supplementary Fig. 9 and simplified in Fig. 1. The photoexcitation of Ru(Phen)_3_ chromophore at 400 nm populates manifold ^1^MLCT states, which should be followed by ultrafast ISC processes into ^3^MLCT triplet states located on Phen part of ligand. The subsequent excited-state relaxation occurs via an ILCT process from the Phen-based state to the BIm-based state, and finally, a much slower kinetic process of LMCT takes place, which realizes the final electron transfer from Ru(Phen)_3_ photocenter to Pd(Py)_4_ catalytic center. The initial picosecond process (*τ*_1_=0.3 ps) should relate to equilibration of Phen-based ^3^MLCT states in a similar time scale as analogous Ru-complexes^49^,^50^,^51^,^52^ and Ru–Pd PMDs^24^, which probably contain contributions from interligand hopping, vibrational cooling and intraligand vibrational relaxation^28^,^30^. As suggested by Huijser *et al*.^28^, two distinct Phen-based ^3^MLCT states may generate, one hot ^3^MLCT state and the other a relaxed state. The ILCT process occurs only from the hot state within 7 ps, which is comparable to a Ru–Pd complex in 4 ps time scale^16^, resulting in the BIm-based excited state. The slow LMCT kinetic process with a characteristic time constant of 122 ps is faster than the Ru–Pd PMD reported by Dietzek (310 ps)^17^, but significantly different from another polypyridyl Ru–Pd complex (*ca.* 100 ns)^28^, which may be owing to presence of multi-channels for directional electron transfer in MOC-16. Noteworthily, analogous LMCT process is absent in free RuL_3_ metalloligand, because the extraordinarily slow decay (>1,000 ps) in RuL_3_ should correspond to the photoluminescence process, albeit the initial (0.2 ps) and ILCT (7 ps) processes in RuL_3_ are similar with MOC-16. It should be reminded that the photophysical processes in heteronuclear PMDs are known to be rather complicated, and another photophysical scheme may also explain the oberved transient absorption data. Note also that not all excited electrons undergo this radiationless deactivation pathway for catalysis, and a competing loss channel to the relaxed ^3^MLCT state caused by vibrational relaxation is possible for radiative decay in the nanosecond domian (Supplementary Fig. 9)^28^. On the basis of the above discussion, we speculate that the photoinitiated directional electron transfers from Ru-photocenters to Pd centres in MOC-16 should undergo proper and uniform photophysical pathways to lead to effective production of H_2_. It is proposed that the final LMCT in analogous Ru–PdCl_2_ system is coupled with one Cl^−^ dissociation and Pd^2+^ reduction^25^; therefore, partial Pd–N dissociation may kinetically associate with the sub-nanosecond LMCT process in the present case. The structural deformation around the Pd center within such fast time scale is probably hindered due to the multiple coordinate bonds formed among the six Pd(Py)_4_ and eight Ru(Phen)_3_ fragments, leading to substantial improvement in the robustness of the MOC-16 cage framework in comparison with its individual mononuclear fragments.

In summary, a heteronuclearly self-assembled [Pd_6_(RuL_3_)_8_]^28+^ cage has been developed as a new potential PMD to unify multiple photosensitizing, electron relay and catalytic units, which exhibits high H_2_ evolution activity and durability. The accessibility of MOC-16 in aqueous solution also makes it an interesting PMD candidate concerning the application in artificial photosynthesis. Moreover, the substitutable NH groups and host-guest chemistry of MOC-16 leave more possibilities to modify cage structure or encapsulating additional photo- or catalytic guests to further improve H_2_ evolving property. Investigations along this line are now in progress in order to fabricate new nanoreactors which make the more effective use of their confined MOC cavities.

## Methods

### Materials and measurements

Unless otherwise stated, all raw materials and solvents were obtained from commercial sources and used without further purification. UV-VIS absorption spectra were tested on a Shimadzu UV-3600 UV-Vis spectrometer. The room steady-state emission spectra and emission lifetime were measured by photoluminescence spectrometer (Edinburgh Instruments Ltd FLS980) equipped with a continuous Xe900 Xenon lamp. Transmission electron microscopy was performed on an FEI Tecnai G2F20S-TWIN (US) operated at 200 kV. ^1^H NMR spectra were recorded on Bruker AVANCE III 400 (400 MHz). Chemical shifts were quoted in parts per million (p.p.m.) referenced to the appropriate solvent peak or 0.0 p.p.m. for TMS.

### Transient absorption spectroscopy

The femtosecond time-resolved absorbance difference spectrometer was equipped with a regenerative Ti:sapphire amplifier laser with 500 Hz repetition (Legend Elite USP HE+, Coherent, 35 fs, 800 nm) as the primary laser source. The output beam was split into two. One with a power of 6 μJ per pulse was focused onto pure water to generate a white light continuum as a probe beam. The other beam was frequency doubled using a 150 μm BBO crystal to generate a 400 nm pump beam, and then passed to the translation stage. A mechanical chopper was employed to modulate the pump repetition frequency to 1/2 the probe repetition rate. The pump and probe pulses were focused to a diameter of 500 and 200 μm, respectively, at the flow cell interface using two plano-concave mirrors. The probe pulse was recorded using a fibre spectrometer (Avantes, AvaSpec_ULS2048L-USB2) in external trigger mode. The polarization of the pump beam was set to the magic angle (54.7°) with respect to the probe beam. The optical path in samples was 5 mm. The pump energy was 2 μJ per pulse, and the pump intensity was 5.1 × 10^14^ photos per cm^2^ per excitation pulse. The reported uncertainties in fit parameters were estimated at 90% confidence limits based on the reproducibility from fitting a number of independently measured transients.

### Electrochemical measurements

The cyclic voltammetry was carried out in CH Instruments Electrochemical Analyzer/Workstation (Model 600B series). Measurements were performed in dry and degassed acetonitrile (MeCN) with a standard three electrods system using a glassy carbon working electrode, platnium counter-electrode and a non aqueous Ag/AgCl reference. The supporting electrolyte was 0.1 M tetrabutylammonium hexafluorophosphate (TBAPF_6_). The scan rate was maintained at 100 mV s^−1^.

### Data availability

Detailed experimental procedures, control experiments and the computational details can be found in the Article and Supplementary Information. All other data are available from the authors on reasonable request.

## Additional information

**How to cite this article:** Chen, S. *et al*. A metal-organic cage incorporating multiple light harvesting and catalytic centres for photochemical hydrogen production. *Nat. Commun.* **7,** 13169 doi: 10.1038/ncomms13169 (2016).

## Supplementary Material

Supplementary Information Supplementary Figures 1-11, Supplementary Tables 1-3 and Supplementary Notes 1-4.
