# Supplementary material for: A metal-organic cage incorporating multiple light harvesting and catalytic centres for photochemical hydrogen production
Source: Nat Commun. 2016 Nov 9;7:13169. doi: 10.1038/ncomms13169 (PMC5105156; doi:10.1038/ncomms13169)
Supplement: Supplementary Information — Supplementary Figures 1-11, Supplementary Tables 1-3 and Supplementary Notes 1-4. [file ncomms13169-s1.pdf]

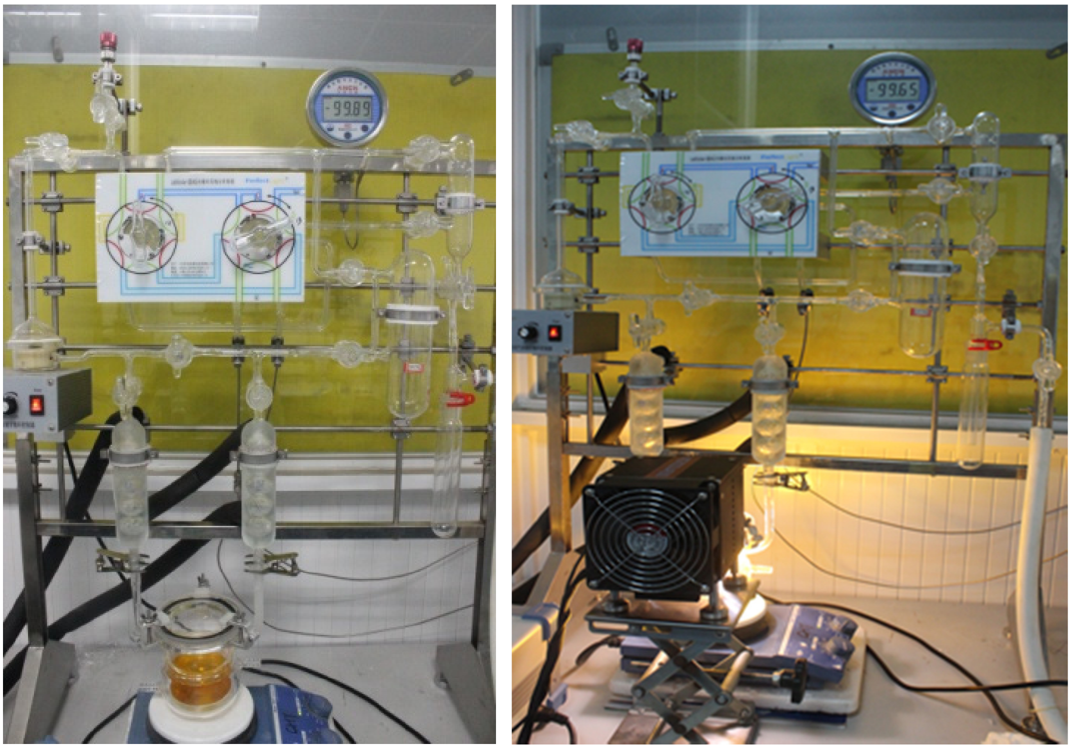

Supplementary Figure 1 | H_2 evolving instrument. A closed gas circulation and evacuation system for H_2 evolution.

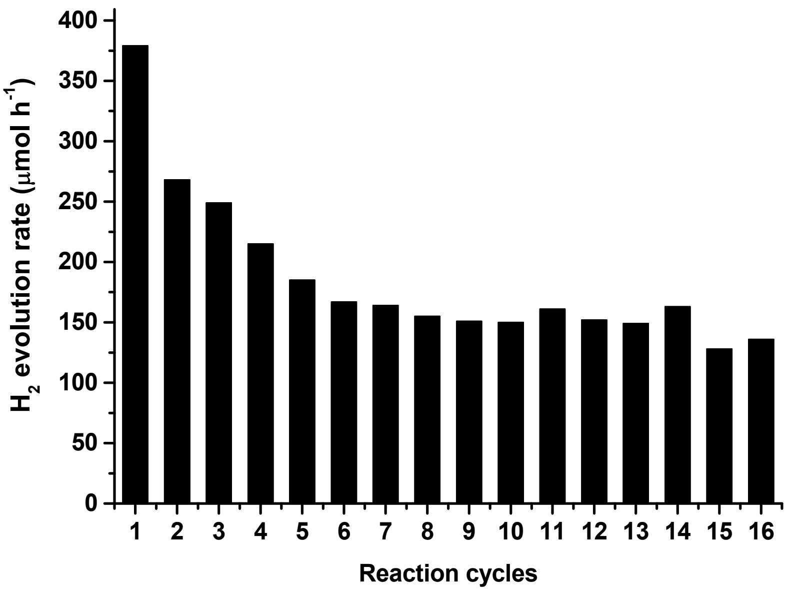

Supplementary Figure 2 | Initial rate comparison. The initial rate of hydrogen generation in 16 three-hour reaction cycles in DMSO with 22 μM **MOC-16**, 0.34 M H_2O and 0.75 M TEOA under irradiation with visible light.

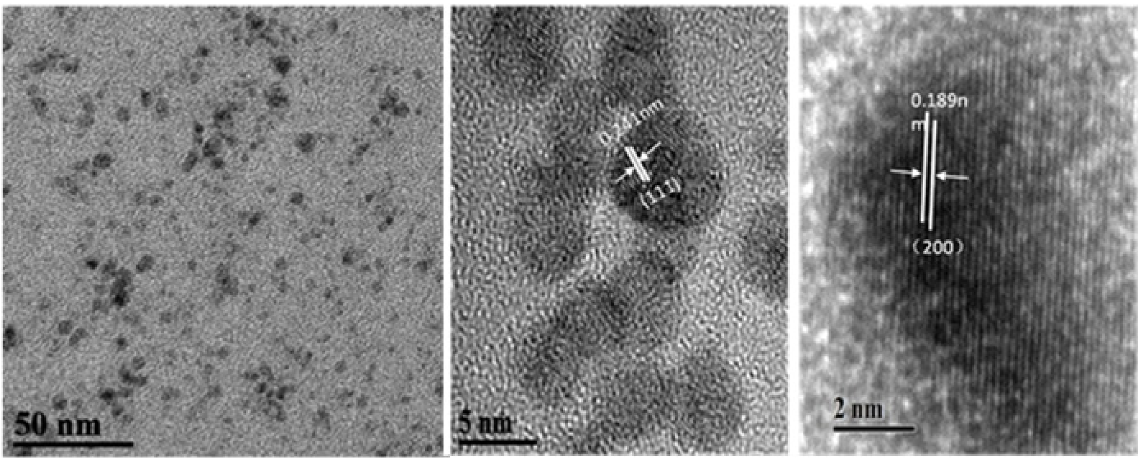

Supplementary Figure 3 | TEM and HRTEM images of Pd nanoparticles obtained after longtime irradiation of the photocatalyst solution (~100 h).

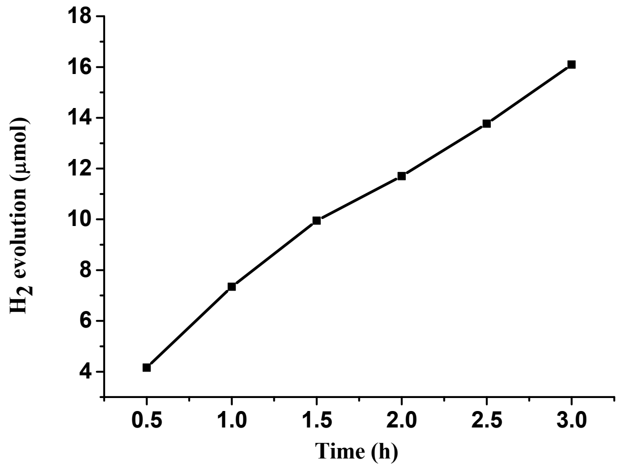

a

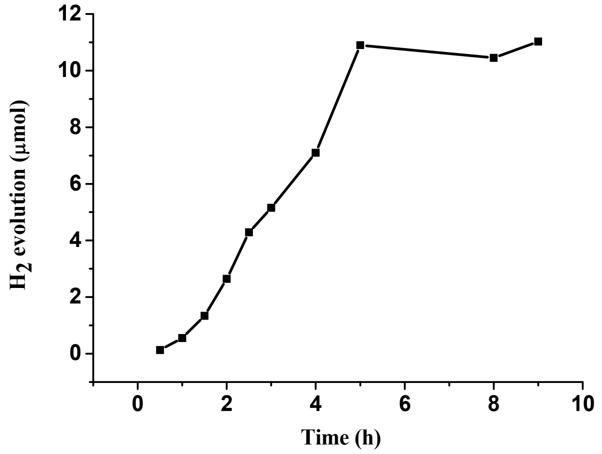

b

Supplementary Figure 4 | H_2 production reactions by directly mixing RuL_3 metalloligand and Pd particles: (**a**) in situ generated Pd particles by irradiating a $Pd(BF_4)_2$ solution of DMSO with UV light overnight; (**b**) commercial Pd(0) black. $C_{RuL3} = 22 \times 8$ μmol L^{-1}, $C_{Pd} = 22 \times 6$ μmol L^{-1}.

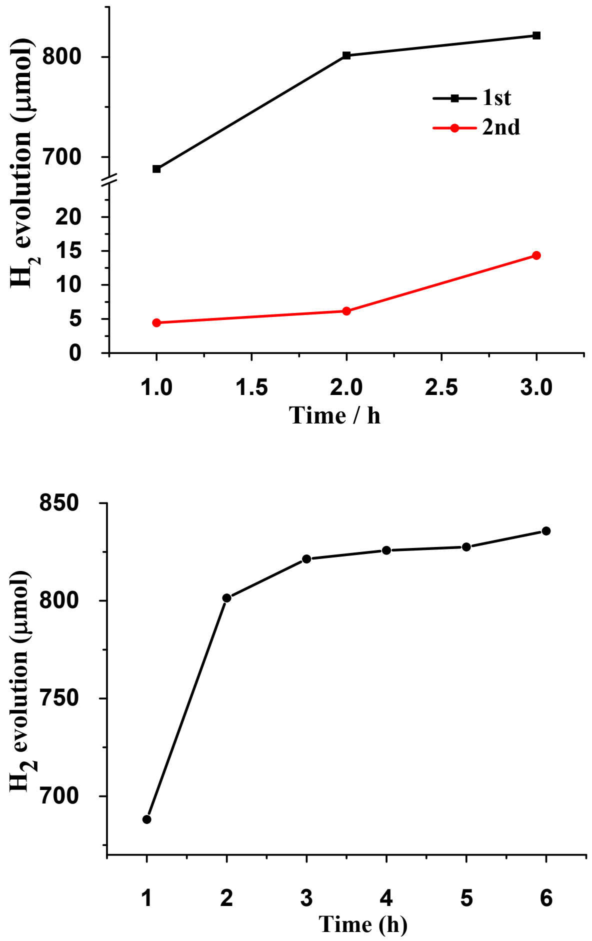

Supplementary Figure 5 | H_2 production reactions by directly mixing $Ru(bpy)_3Cl_2$ with $Pd(Py)_4(BF_4)_2$. $C_{Ru(bpy)3Cl2} = 22 \times 8$ µmol L^{-1}, $C_{Pd(Py)4(BF4)2} = 22 \times 6$ µmol L^{-1}.

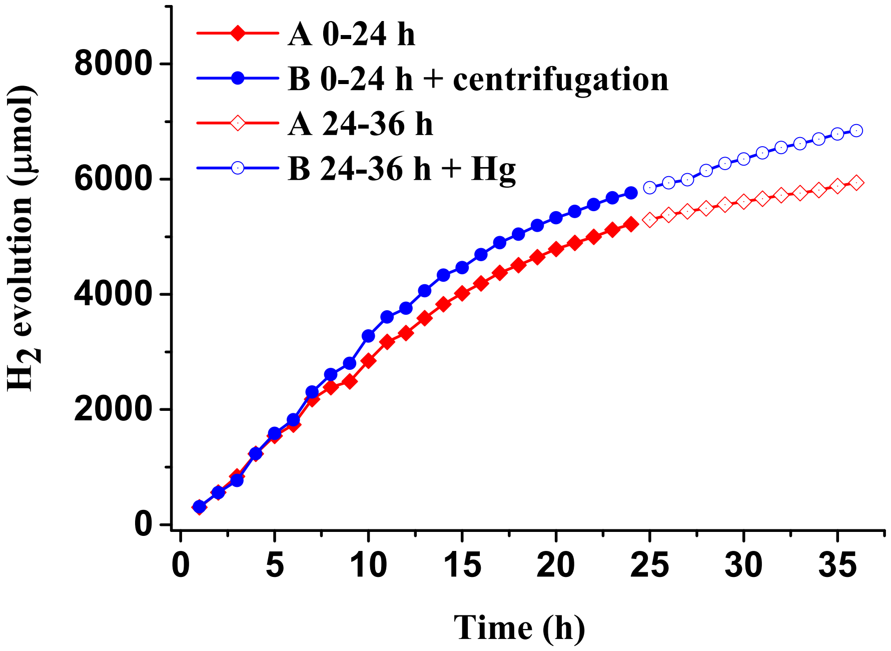

Supplementary Figure 6 | H_2 production from two parallel reactions in total 36 h for each: **Reaction A**. consecutive 12 three-hour reaction cycles (red line) without any further treatment; **Reaction B**. consecutive 12 three-hour reaction cycles (blue line) with centrifugation of reaction solution after every three-hour run from 0 to 24 h and Hg-test for possible catalyst poisoning after every three-hour run from 24 to 36 h.

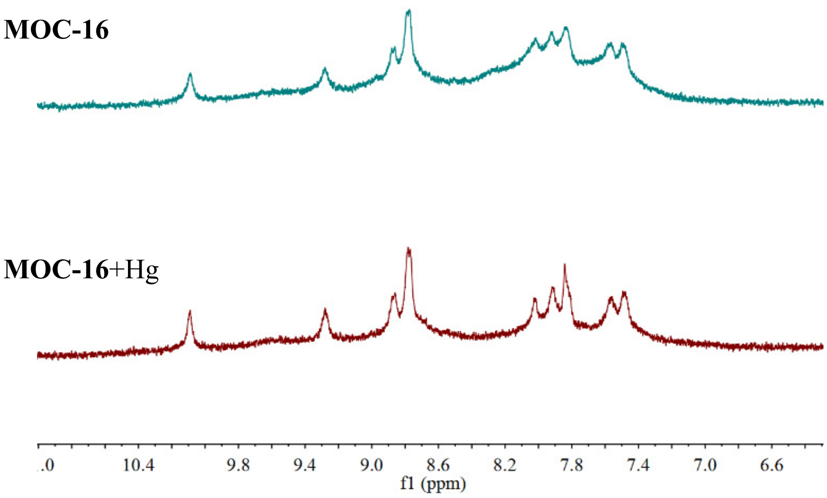

Supplementary Figure 7 | ^{1}HNMR spectra (400 MHz) of **MOC-16** before and after 9 h stirring with Hg in DMSO-d_6/D_2O (V:V=1:10).

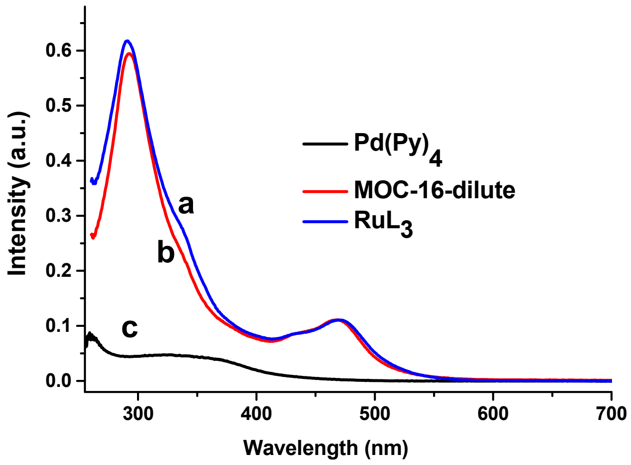

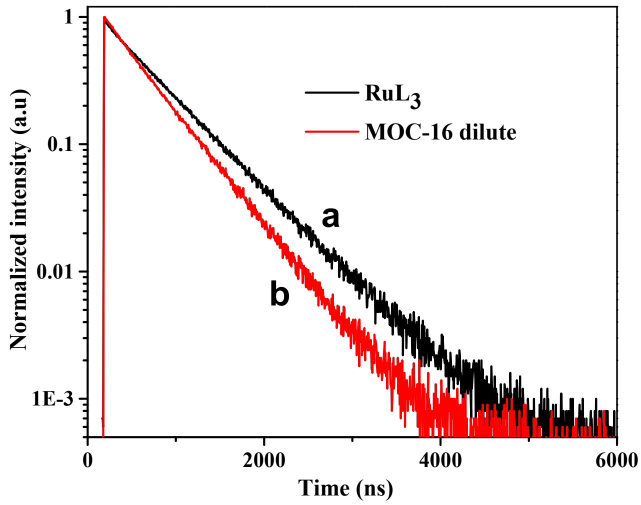

Supplementary Figure 8 | Electronic absorption spectra of (**a**) RuL_3 (**C_{RuL3}** = 8 × 10^{-6} M), (b) **MOC-16-dilute** (**C_{MOC-16}** = 0.6 × 10^{-6} M) and (**c**) $Pd(Py)_4$ ($C_{Pd(py)4}$=6 ×10^{-6} M) (upper). **Time-resolved emission decay** at 610 nm of (**a**) RuL_3 (C_{RuL3} = 1.76 × 10^{-4} M) and (**b**) **MOC-16-dilute** ($C_{MOC\text{-}16\ dilute}$ = 1.32 ×10^{-5} M) in DMSO (lower).

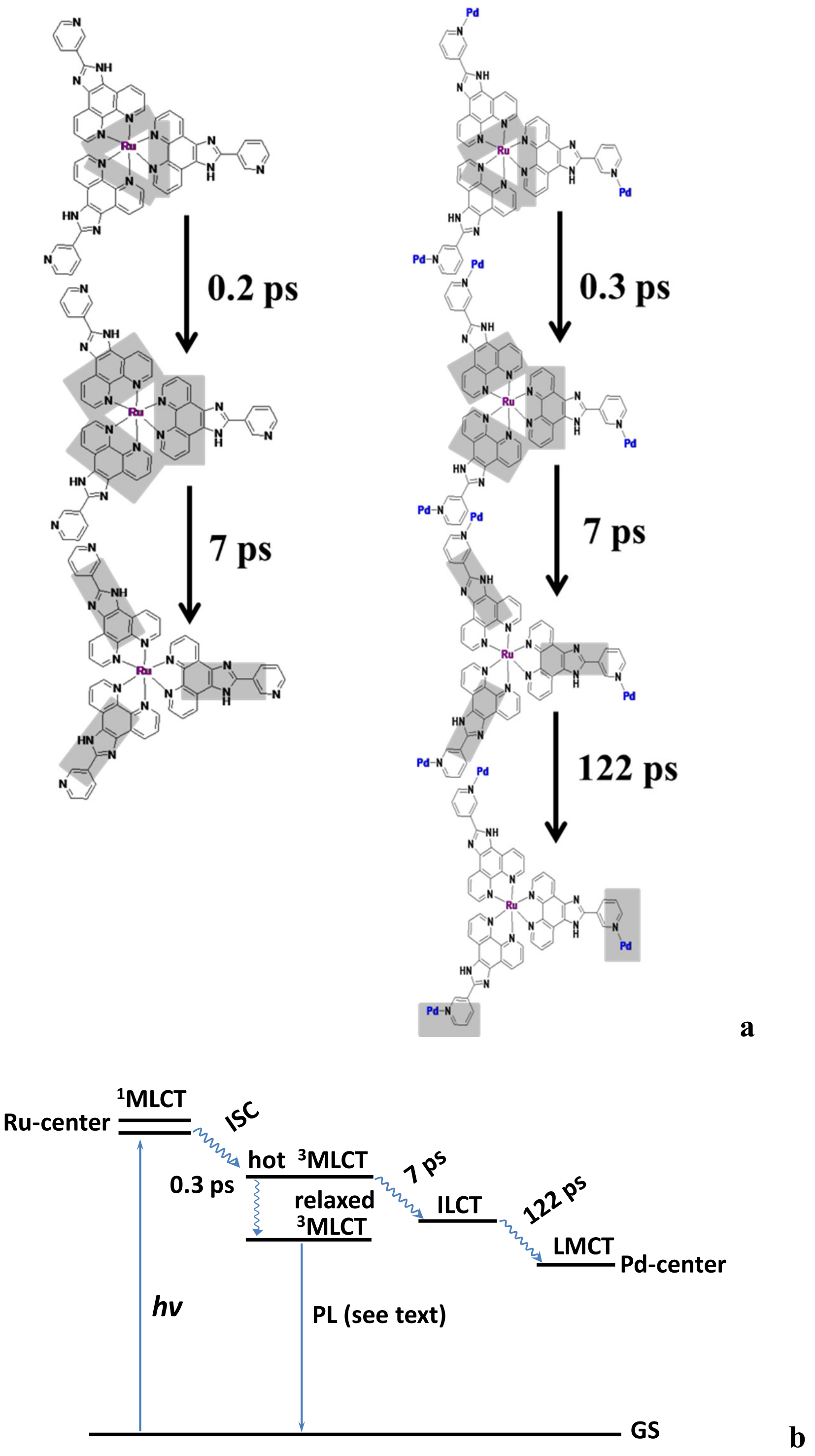

Supplementary Figure 9│Possible light-induced photophysical processes. (a) Localization of the photoexcited electron within the supramolecular unit highlighted by shaded areas in RuL_3 metalloligand and **MOC-16** (showing one eighth part). (**b**) Simplified photophysical model for photoinduced electron transfer in **MOC-16**. MLCT = metal-ligand charge transfer, ILCT = intra-ligand charge transfer, LMCT = ligand-to-metal charge transfer, GS = ground state, PL = photoluminescence, ISC = intersystem crossing. Only major processes most likely for energy and electron transitions between chromophoric-Ru and catalytic-Pd metal centers are shown.

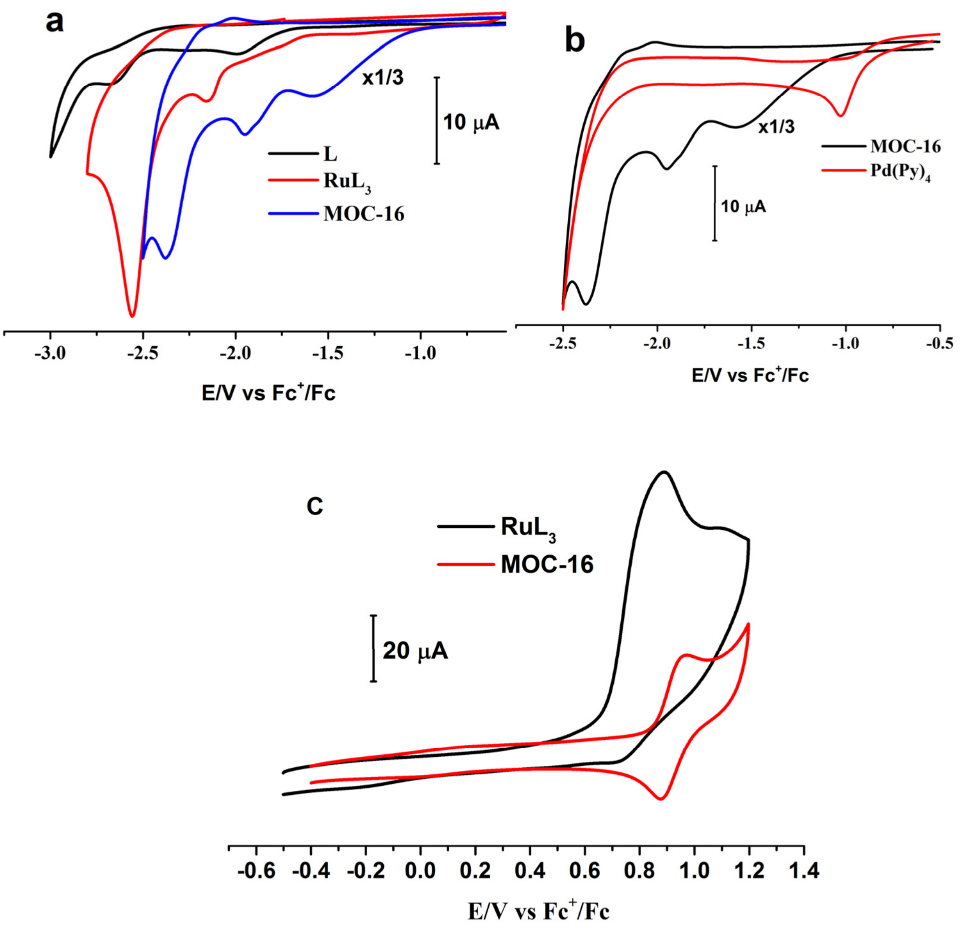

Supplementary Figure 10 | Cyclic voltammogram comparison of **MOC-16** (0.125 mM), RuL_3 (1 mM), PhenBImPy ligand (1 mM) and $Pd(Py)_4$ (1 mM) in CH_3CN (rt, sweep = 100 mV s^{-1}, non-aqueous Ag/AgCl electrode as reference, 0.1 M $TBAPF_6$ as supporting electrolyte).

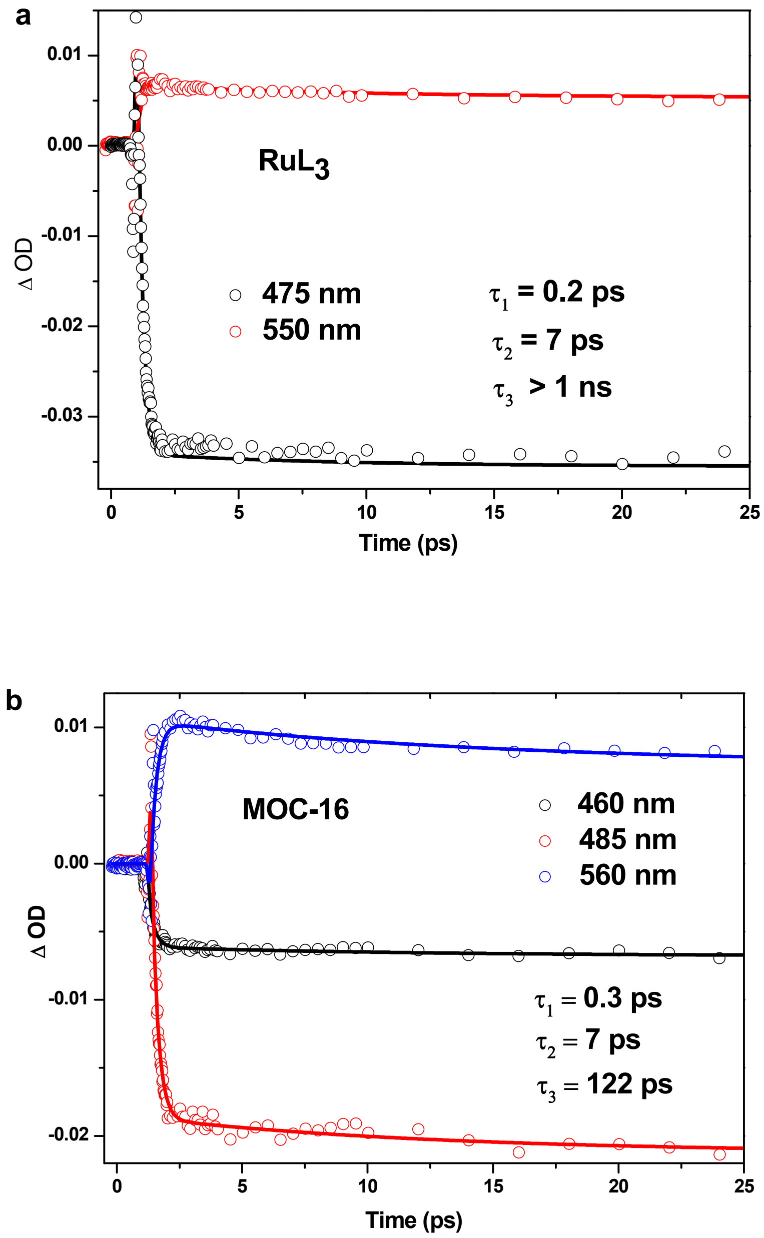

Supplementary Figure 11 | Kinetic traces over the first 25 ps at different probe wavelengths of (**a**) RuL_3 and (**b**) **MOC-16** in DMSO.

Supplementary Table 1 | Photoluminescence and transient absorption lifetimes of RuL_3 and **MOC-16** in DMSO.

**Sample**	**PL Lifetime/ ns**	**TA lifetime/ ps**		
	τ	τ_1	τ_2	τ_3
RuL_3	601	0.2	7	>1000
**MOC-16**	484	0.3	7	122

Supplementary Table 2 | Frontier molecular orbitals of a fully optimized structure of $Pd_6(RuL_3)_8$ cage in vacuum, obtained by using the B3LYP level of DFT and the 6-31G/LanL2DZ basis set implemented in the Gaussian 09 suite programs.

**LUMO+10**	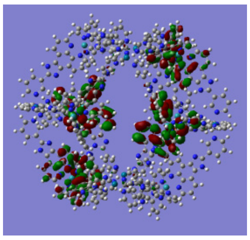	**LUMO+6**	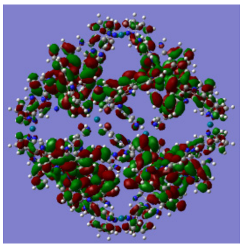
**LUMO+9**	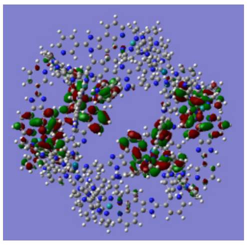	**LUMO to LUMO+5**	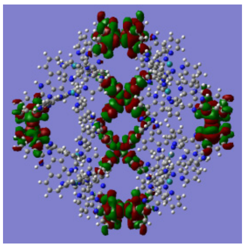
**LUMO+8**	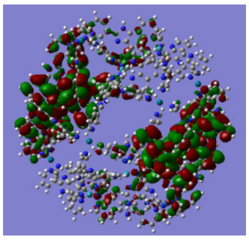	**HOMO to HOMO-7**	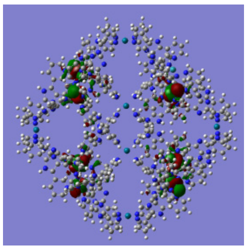
**LUMO+7**	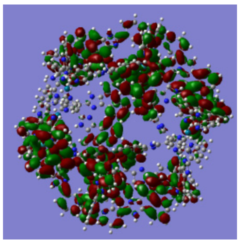		

Supplementary Table 3 | Electrochemical data in acetonitrile.[a]

Complex	$E_{1/2}$(ox), V	E_p (red), V
**L**	-	-2.06, -2.74
**RuL$_3$**	0.90	- -2.15, -2.57
**Pd(Py)$_4$**	-	-1.04--
**MOC-16**	0.92	-1.58,-1.95,-2.38

[a]All potentials are referenced to an Fc/Fc$^+$ couple.

Supplementary Note 1. Synthesis of RuL_3 and MOC-16 complex

Preparation of $RuL_3(BF_4)_2$. Procedure for preparation of metalloligand **RuL_3** follows our previous work.[38] The identity of prepared **RuL_3** was checked by comparison of ^{1}HNMR data with the reported ones.

Preparation of cages $[Pd_6(RuL_3)_8]X_{28}$•solvents ($X = BF_4^-$). The cage **MOC-16** was prepared also according to above literature. Typically, 13 mg $RuL_3(BF_4)_2$ (0.01 mmol) and 3 mg $Pd(BF_4)_2(CH_3CN)_4$ (0.0075 mmol) were dissolved in 0.5 ml DMSO. The mixture was heated at 80℃ for overnight with stirring. 3 ml ethylacetate was added after reaction finished. The precipitates were centrifuged, washed with ethylacetate, and dried to get solid. The identity of prepared **MOC-16** was checked by comparison of ^{1}HNMR and MS data with the reported ones.

Preparation of $Pd(Py)_4(BF_4)_2$. Pyridine (200 μL) was added to $Pd(BF_4)_2(MeCN)_4$ (180 mg, 0.4 mmol) in MeCN (5 mL) and stirred at 60 °C for 6 h. After cooling to room temperature, diethyl ether was added to this solution and stirred, and then the solvent was removed by centrifugation. The precipitates was ultrasonic rinsed and dryed to get the product. The identity of prepared **$Pd(Py)_4(BF_4)_2$** was checked by ^{1}HNMR data and ESI MS. **$[Pd(Py)_4(BF_4)]^+$** *m/z*: 509.0757.

Supplementary Note 2. Photocatalytic hydrogen production

Hydrogen production experiments were carried out using a closed gas circulation and evacuation system. The amount of produced hydrogen was analyzed using an on-line gas chromatography (Agilent 7820A with a thermal conductivity detector and a N_2 carrier). Typically, 0.030g powder sample was dissolved in 100 mL mixed solution in a Quartz reaction cell with magnetic stirring. Each sample was made up with dimethyl sulfoxide (DMSO) as solvent, H_2O as the proton source, and

triethanolamine (TEOA) as the sacrificial reducing agent. C_{TEOA} = 0.75 mol L^{-1}, $C_{complex} = 2.2 \times 10^{-5}$ mol L^{-1}. The light source was a 300 W Xe lamp (PLS-SXE-300C, Beijing Perfect light) supplying the visible light (> 420 nm) by using a 420 nm cut-off filter.

Supplementary Note 3. Control experiments

Hydrogen evolving with mixed RuL_3 and Pd particles. The control experiments by directly mixing free RuL_3 metalloligand with Pd particles were performed under the similar reaction conditions, where the concentration of RuL_3 (22 × 8 μM) and Pd (22 × 6 μM) are equivalent to the amount of **MOC-16**. The palladium source is from commercial Pd(0) black, or in situ generated Pd particles by irradiating a $Pd(BF_4)_2$ solution of DMSO with UV light overnight. Only trace amount of H_2 is detected in these two control experiments.

Hydrogen evolving with mixed $Ru(bpy)_3Cl_2$ and $Pd(Py)_4(BF_4)_2$. The control experiments by directly mixing free $Ru(bpy)_3Cl_2$ with $Pd(Py)_4(BF_4)_2$ were performed under the similar reaction conditions, where the concentration of $Ru(bpy)_3Cl_2$ (22 × 8 μM) and $Pd(Py)_4(BF_4)_2$ (22 × 6 μM) are equivalent to the amount of **MOC-16**. A high H_2 evolution rate is found at initial period of reaction, but remarkably declining 2 h later. In the second run, only trace amount of H_2 is detected. Meanwhile, black Pd appears in this reaction mixture, which indicates decomposition of $Pd(Py)_4(BF_4)_2$ to Pd particles.

Parallel reactions with interval centrifugation and mercury test. To check if Pd particles are formed at early stage and elucidate the role of Pd colloids in the photocatalyzed H_2 production, two parallel reactions are carried out in 100 mL DMSO solution containing 22 μM **MOC-16**, 0.34 M H_2O and 0.75 M TEOA under irradiation with visible light. **Reaction A**: Photoinduced H_2 production was performed consecutively for 36 h in total based on 12 cycles of three-hour reaction (12 × 3h) without any treatment of the reaction mixture. **Reaction B**. Photoinduced H_2 production was performed consecutively for 36 h in total also based on 12 cycles of

three-hour reaction. However, in the first 0-24 hours of reaction, the reaction mixture was centrifugated after every three-hour run with Allegra 64R Centrifuge at a speed of 13000 rpm for 10min. In the remaining reaction time from 24 to 36 h, H_2 production was performed in the presence of mercury for catalyst poisoning test. Before 9th run of reaction, 200 μL Hg was added to the reaction mixture (Pd:Hg = 13.2 μmol:1mmol). To make sure high dispersion of Hg in reaction solution for full contact with **MOC-16**, the mixture was constantly and vigorously stirred during reaction. At each reaction interval from 9th to 12th runs, the mixture was further violently shaken to guarantee a sufficient mixing of Hg phase and DMSO phase.

The accumulated H_2 production from both **A** and **B** reactions is depicted in Supplementary Fig. 6. It is evident that the photocatalyzed reaction is not obviously affected by the treatments of centrifugation and Hg-test at least in 36h. In the first 0-24 h reaction, no observable Pd particles were isolated from centrifugation.

Cage integrity against Hg tested by ^{1}H NMR. To check if the cage structure will be destructed by Hg, we measured ^{1}HNMR spectra of **MOC-16** (Ru_8Pd_6) after vigorously stirring with Hg. A solution of 0.5 mL DMSO-d_6 containing 15 mg (1.1 μmol) **MOC-16** in the presence of 3.1 mL liquid mercury (Pd:Hg=6.6 μmol:16 mmol) was stirred vigorously for 9 h without irradiation and addition of triethanolamine. Afterwards, the supernatant **MOC-16** solution was filtered through a syringe filter. About 50 μL reaction solution was added into the NMR tube to mix with 450 μL D_2O. The obtained ^{1}HNMR spectrum shows no significant signal change in comparison with that of fresh MOC-16.

Supplementary Note 4. Preliminary calculation on frontier orbitals.

To depict the frontier orbitals, a preliminary calculation on **MOC-16** cage based on the structural model from its single-crystal data[38] has been performed with B3LYP hybrid functional. The all electron basis set, 6-31G, was employed in nonmetallic elements, while the pseudopotential basis set, LanL2DZ, was used in Ru and Pd. The structure optimized by semi-empirical method PM6 was subsequently manipulated by

the much expensive density functional method. All the calculations were performed on Gaussian09 package. Due to the fact that the whole **MOC-16** cage molecule is too large and a full calculation of this structure model is tremendously time-consuming, TDDFT calculation has not been finished at this stage. In this article, we only focus on the distribution of the related frontier orbitals.
